# Flagella disruption in *Bacillus subtilis* increases amylase production yield

**DOI:** 10.1186/s12934-022-01861-x

**Published:** 2022-07-02

**Authors:** Annaleigh Ohrt Fehler, Thomas Beuchert Kallehauge, Adrian Sven Geissler, Enrique González-Tortuero, Stefan Ernst Seemann, Jan Gorodkin, Jeppe Vinther

**Affiliations:** 1 Section for Computational and RNA Biology, Department of Biology, University of Copenhagen, Copenhagen, Denmark; 2 Novozymes A/S, Copenhagen, Denmark; 3 Center for non-coding RNA in Technology and Health, Department of Veterinary and Animal Sciences, University of Copenhagen, Copenhagen, Denmark

**Keywords:** Bacillus subtilis, Industrial production, CRISPR-dCas9, Flagella, Motility

## Abstract

**Background:**

*Bacillus subtilis* is a Gram-positive bacterium used as a cell factory for protein production. Over the last decades, the continued optimization of production strains has increased yields of enzymes, such as amylases, and made commercial applications feasible. However, current yields are still significantly lower than the theoretically possible yield based on the available carbon sources. In its natural environment, *B. subtilis* can respond to unfavorable growth conditions by differentiating into motile cells that use flagella to swim towards available nutrients.

**Results:**

In this study, we analyze existing transcriptome data from a *B. subtilis* α-amylase production strain at different time points during a 5-day fermentation. We observe that genes of the *fla/che* operon, essential for flagella assembly and motility, are differentially expressed over time. To investigate whether expression of the flagella operon affects yield, we performed CRISPR-dCas9 based knockdown of the *fla/che* operon with sgRNA target against the genes *flgE, fliR,* and *flhG*, respectively. The knockdown resulted in inhibition of mobility and a striking 2–threefold increase in α-amylase production yield. Moreover, replacing *flgE* (required for flagella hook assembly) with an erythromycin resistance gene followed by a transcription terminator increased α-amylase yield by about 30%. Transcript levels of the α-amylase were unaltered in the CRISPR-dCas9 knockdowns as well as the *flgE* deletion strain, but all manipulations disrupted the ability of cells to swim on agar.

**Conclusions:**

We demonstrate that the disruption of flagella in a *B. subtilis* α-amylase production strain, either by CRISPR-dCas9-based knockdown of the operon or by replacing *flgE* with an erythromycin resistance gene followed by a transcription terminator*,* increases the production of α-amylase in small-scale fermentation.

**Supplementary Information:**

The online version contains supplementary material available at 10.1186/s12934-022-01861-x.

## Background

In its natural environment of soil and plant rhizosphere, *Bacillus subtilis* feed by secreting large amounts of enzymes to metabolize available biomass. In combination with an effective fermentation, this optimized secretion system has made *B. subtilis* and related Bacilli the natural choice for industrial production of protein [9, 36, 40]. Over the years, production strains have been further improved by genetic modification to increase yield. Protein secretion has been optimized by overexpression of the chaperone PrsA [5, 17, 30] and extracellular proteases have been deleted to limit recombinant protein degradation [29, 34]. Cell factories based on *B. subtilis* typically use a fed-batch setup, where carbon is continuously supplied to the cells and the protein product accumulates over time in the supernatant. In the early phase of fermentation, cell mass will increase, whereas it will be constant or even reduce slightly in the later stages, likely predominantly because of nutrient starvation.

*B. subtilis* is adapted to survival in soil, which in most cases is a very nutrient-poor environment. Thus, *B. subtilis* can differentiate into different specialized cell types, which will improve chances for survival during starvation [20, 21]. Most dramatic is the formation of endospores by an asymmetric cell division, where the bacterial genome is packaged in a multilayered protein coat, which can protect it for extended periods of time and allows the bacteria to dwell until conditions improve. For cell factories, spore formation is unwanted because it is energy expensive and cause contamination of products. Thus, the *B. subtilis* production strains used in this work have inactivated spore formation. Sporulation is the last resort in response to different types of stress, including nutrient starvation. Before the cells commit to sporulation, they will typically try to adapt to environmental changes by processes such as cannibalism, which involves selective killing of sister cells to provide nutrients or the acquirement of motility to allow cells to seek out an environment more suitable for growth and survival [20, 21]. *B. subtilis* cells can both swim in liquid solutions and swarm over solid surfaces towards more favorable conditions. The movement is made possible by rotating flagella that are complex machineries of more than 30 proteins and covers motile cells in a peritrichous arrangement [13]. The flagella consist of long protein filaments of the protein Hag attached to a hook structure composed of FlgE. The hook structure is in turn anchored to a basal body in the membrane that serves as a platform for assembly in flagella synthesis. The majority of proteins comprising the basal body are encoded in the 26.7 kb *fla/che* operon, where FliH, FliI, FliJ, FliO, FliP, FliQ, FliR, FlhA, and FlhB constitute a type III secretion system which secretes proteins that form the hook and filament structures [23].

Each motile *B. subtilis* cell has over 20 flagella basal bodies, and the process of flagella assembly takes more than 40 min with filament polymerization being the rate-limiting step, meaning that in rapidly growing cells, flagella will be formed over multiple generations [13]. The process of flagella assembly is not only slow but also highly energy expensive and therefore subject to extensive regulation. The decision of whether to adopt a motile state is determined by the regulation of the *fla/che* operon, which also encodes the alternative sigma factor SigD, which can further activate genes involved in filament polymerization and rotation [26]. SigD serves as an ON/OFF switch for the decision to become motile, with motile cells having a high level of expression whereas non-motile cells have a low level [16]. The *fla/che* operon is controlled by two promoters, one driven by the housekeeping sigma factor, SigA, and the other being SigD-dependent, which creates a positive feedback loop for SigD. In addition, SigD is inhibited by binding the anti-sigma factor FlgM [4], which is secreted via the flagella upon hook-basal body completion, thereby potentially contributing to the bistable expression pattern of SigD [3]. The SigD-dependent promoter for the flagella operon is furthermore repressed by binding heterodimers of the SlrR and SinR factors [7]. For cells to swarm, hyperexpression of flagella is necessary and central for this process is SwrA, which together with the hyperphosphorylated form of DegU binds and derepresses the *fla/che* operon [16, 25]. In this way, SwrA contributes to setting the threshold for activation of SigD [16]. Most laboratory strains harbor mutations in SwrA that biases cells towards a sessile state [27]. In the absence of SwrA, the hyperphosphorylated form of DegU inhibits *fla/che* operon expression [25]. Interestingly, DegU mutants that are unable to bind to SwrA and have increased stability of the phosphorylated form show the so-called Hy phenotype characterized by the absence of flagella and hyperproduction of extracellular proteases and amylases [2, 8].

Despite the importance of flagella driven motile behavior in the wild, it remains unknown whether this behavior is important for fed-batch fermentation yield. With outset in the transcriptome analysis showing that the genes in the *fla/che* operon have significant differences in expression levels over time [11], we here focus on this operon and investigate how flagella disruption affects the yield of enzyme production using a *B. subtilis* production strain and demonstrate that it significantly increases the yield of α-amylase production in *B. subtilis*.

## Results

### Regulation of the fla/che operon during fermentation

**Fig. 1:**
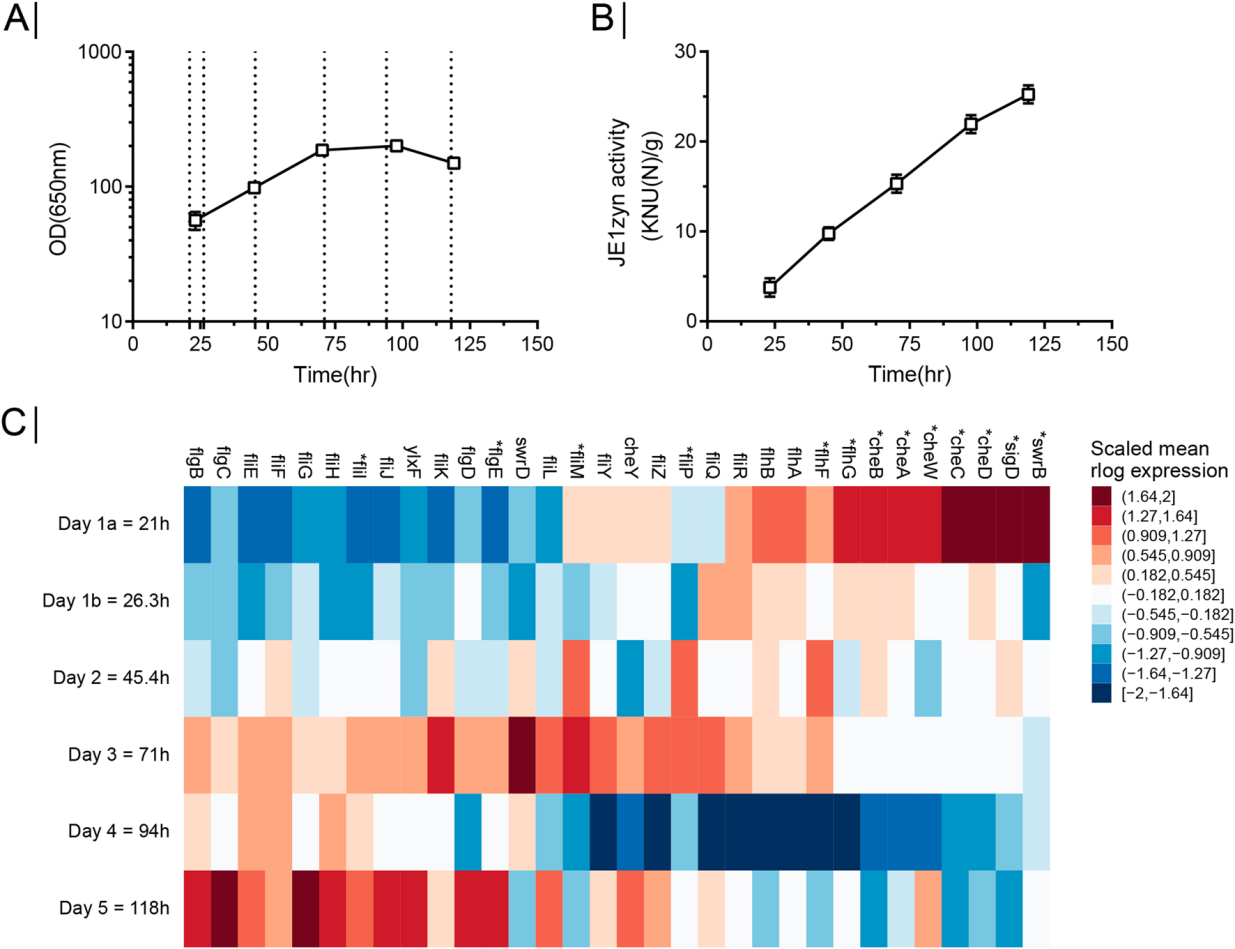
Expression dynamics of flagella operon during fermentation. **A** Culture density during fermentation measured at OD_650_ in triplicates. Error bars depict standard deviation. Dashed line marks sampling time points for RNA-seq **B** Protein yield measured as JE1zyn activity during fermentations in triplicates. Error bars depict standard deviation. **C** Heatmap of the temporal expression dynamics across the 32 genes of the flagella operon showing the per gene z-scaled mean of DESeq2’s rlog transformed data. 13 genes (marked with stars) had statistically significant changes in expression levels during the fermentation according to a test with DESeq2 (FDR adjusted p ≤ 0.05, 5 comparisons along the time axis and 4 against expression at first time-point, see Additional File 1: Figure S1 and S2). The columns are sorted according to their position within the flagella operon

We recently investigated RNA expression during fed-batch fermentation of a *B. subtilis* α-amylase producing strain using RNA-Seq [11]. The RNA expression of three biological replicates was investigated at 6 time points covering the late phase of cell mass increase and the stationary phase (Fig. 1A) while the yield of the α-amylase JE1zyn increased over the entire fermentation period (Fig. 1B). Working with the global expression dataset, we noticed that the expression for genes in the *fla/che* operon changed over the time course of the fermentation. The expression levels for genes in the first half of the operon increased over time, while those in the last part of the operon were expressed at a higher level on the first day compared to later days in fermentation (Fig. 1C) with 13 of the 32 genes in the operon found to be significantly differentially expressed over time (See Additional File 1: Figure S1 and S2). SigD, SwrB, and the chemotaxis proteins are important regulators of flagella [24, 32, 37] and their increased expression in early fermentation points towards acquirement of motility at least for some of the cells.

### CRISPR-dCas9 based knockdown of the fla/che operon

**Fig. 2:**
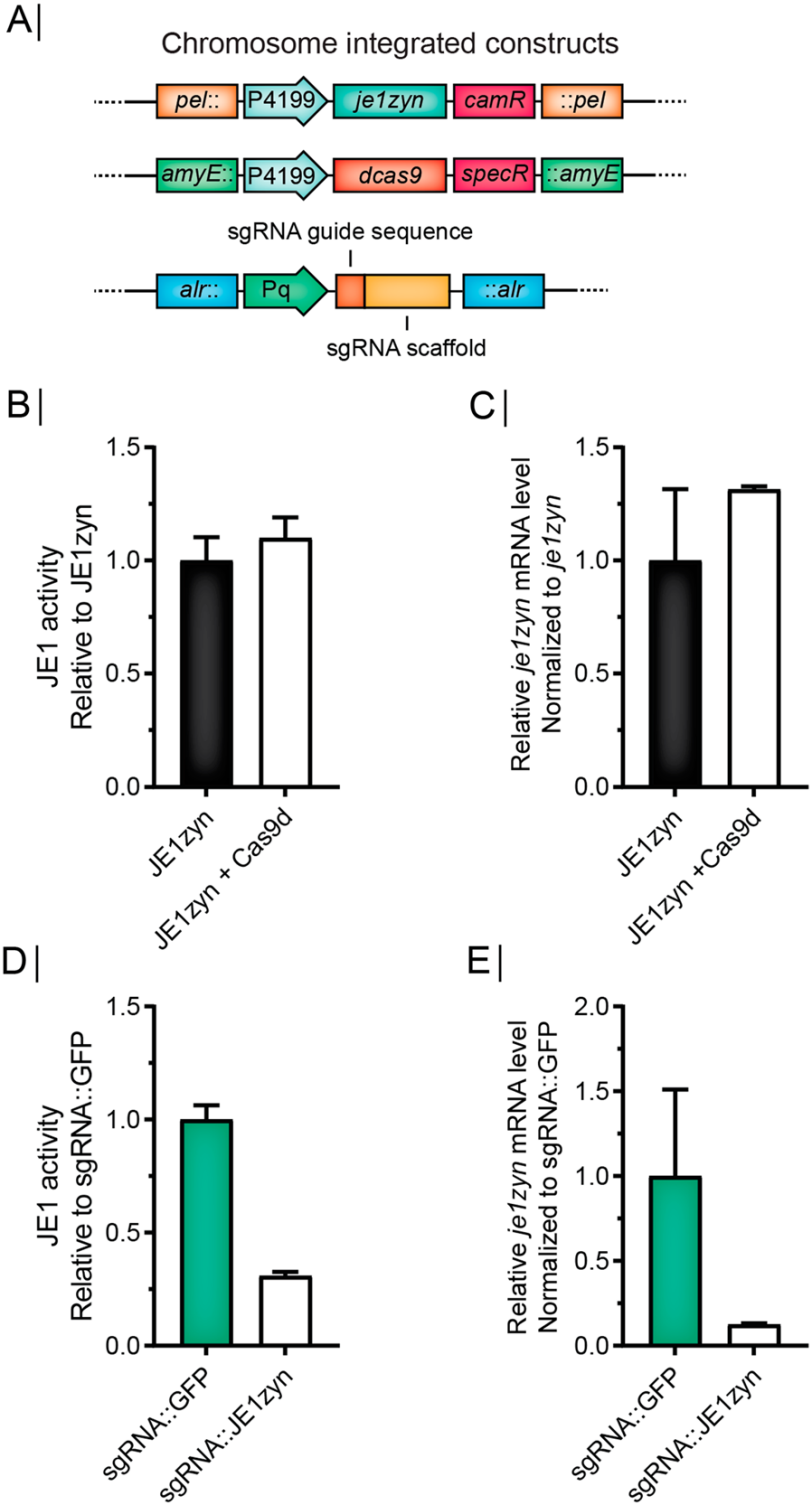
CRISPR-dCas9 setup in JE1-producing strains. **A** Schematic overview of chromosome integrated constructs in strains used for CRISPRi. *je1zyn* is a synthetic gene encoding an α-amylase placed upstream to chloramphenicol acetyltransferase (*cat*) that allow chloramphenicol resistance selection. Dead Cas9 (dCas9) binds specific DNA regions based on sequence complementarity of the single guide RNA (sgRNA) to block transcription at said region. Spectinomycin selection is allowed by insertion of a *specR* gene. Both dCas9 and JE1zyn are expressed from a strong promoter (P4199) [15]. *je1zyn* and dCas9 are inserted in the *pel* and *amyE* loci, respectively. Single guide RNAs are expressed from a Pq promoter and inserted in the *alr* locus. **B** Biolector fermentation JE1 activity of strains expressing JE1zyn and JE1zyn + dCas9 relative to JE1zyn. N = 3, error bars depict standard deviation. **C** qRT-PCR *je1zyn* mRNA levels of strains expressing JE1zyn and JE1zyn + dCas9 relative to JE1zyn. N = 3, error bars depict standard error of the mean. (**D**) as (**B**) but strains expressing JE1zyn + dCas9 + sgRNA::*gfp* (sgRNA::GFP) and JE1zyn + dCas9 + sgRNA::JE1zyn (sgRNA::JE1zyn). (**E**) as (**C**) but strains expressing JE1zyn + dCas9 + sgRNA::*gfp* (sgRNA::GFP) and JE1zyn + dCas9 + sgRNA::*je1zyn* (sgRNA::JE1zyn)

**Fig. 3:**
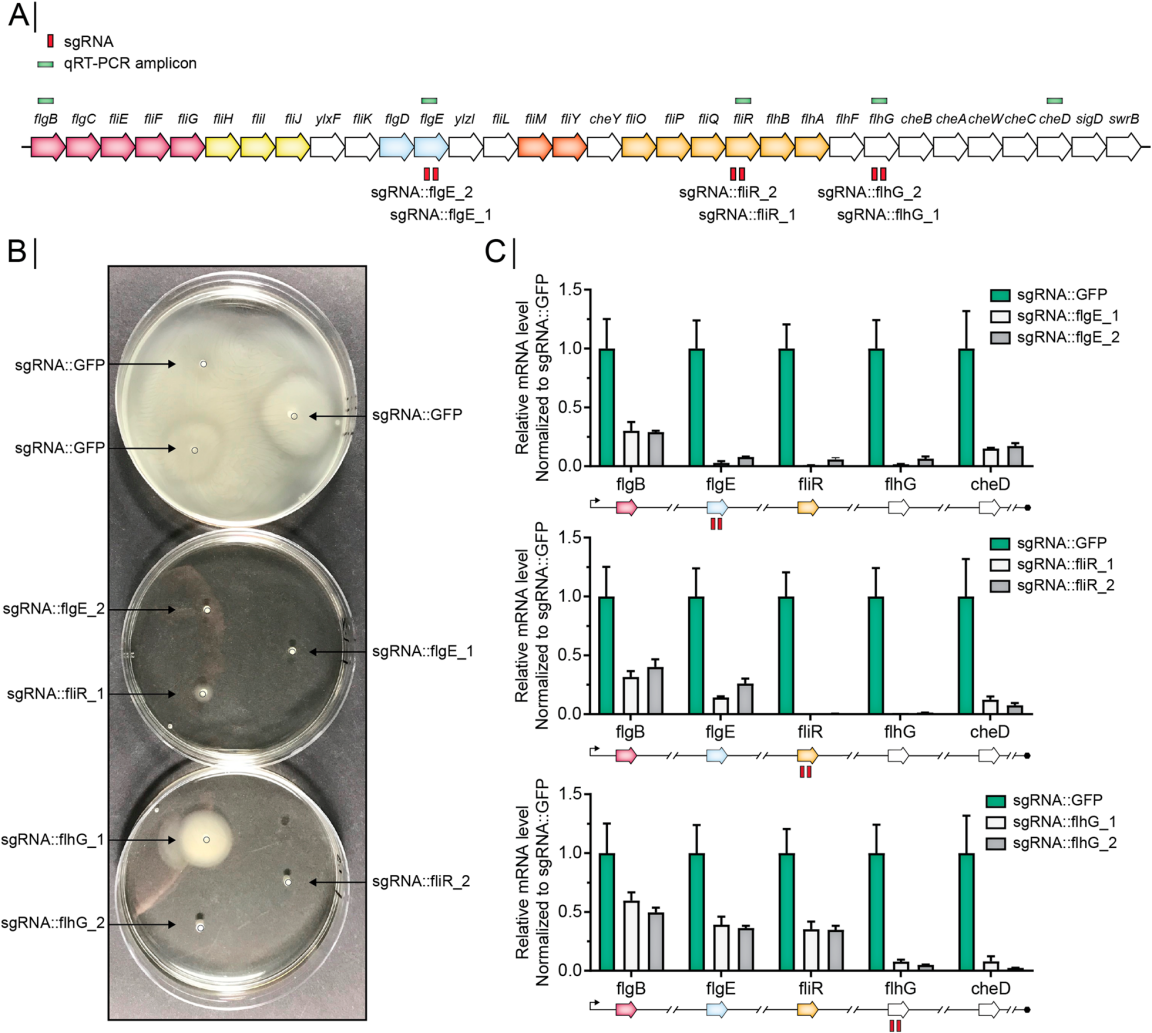
Motility is disrupted by CRISPR-dCas9 targeting of the *fla/che* operon. **A** The *fla/che* operon showing sgRNAs against *flgE*, *fliR,* and *flhG* (red boxes) and qRT-PCR amplicons (green boxes). Gene colors adapted from Mukherjee and Kearns [26] according to gene function. **B** Swimming assay of strains expressing sgRNA against flagellar genes or *gfp*. **C** qRT-PCR of mRNA of flagellar genes (green boxes in **A**) in strains expressing sgRNA::*flgE*, sgRNA::*fliR* or sgRNA::*flhG* (two sgRNAs per gene, grey and white bars) or sgRNA::*gfp* (green bars) normalized to sgRNA::*gfp*. Schematic illustration of *fla/che* operon according to (**A**) is shown below plots. N = 3, error bars depict standard error of the mean

To investigate how a reduced expression of the *fla/che* operon affected the yield of heterologous protein expression, we employed a CRISPR-dCas9 based system that allows the recruitment of a catalytically inactive Cas9 to a DNA region of interest by co-expression of a sequence-specific single guide RNA (sgRNA) leading to a block in transcription [19, 28]. We constructed a strain that expresses dCas9 and an α-amylase (JE1) from a codon-optimized expression cassette *je1zyn* (Fig. 2A), thereby allowing the effect of gene knockdown on yield to be evaluated through sgRNA co-expression. First, we established that the expression of dCas9 affected neither the activity of JE1 in small-scale fermentations (Fig. 2B) nor the expression of *je1zyn* mRNA (Fig. 2C). Next, we tested a sgRNA targeted to *je1zyn*, which as expected reduced JE1 enzyme activity to 30.9% (Fig. 2D) and *je1zyn* mRNA to 12.6% (Fig. 2E) compared to the levels observed for a sgRNA targeted against a control *gfp* sequence. This shows that our CRISPR-dCas9 setup leads to a partial knockdown of expression rather than a complete knockdown of targeted genes. We then employed the CRISPR-dCas9 to perform partial knockdown of flagella by targeting three different genes in the *fla/che* operon. For each of the genes (*flgE, fliR,* and *flhG*), we used two different sgRNAs (Fig. 3A). Compared to a control strain expressing a sgRNA directed against *gfp,* swimming was disrupted or severely repressed for all six strains (Fig. 3B), suggesting that the expression of the sgRNAs resulted in downregulation of the operon. This was validated by performing qRT-PCR of 5 loci in the *fla/che* operon (Fig. 3A and C). Indeed, we found that dCas9 targeting efficiently inhibits expression, not only of the genes targeted by sgRNAs, but also other genes in the operon downstream of the sgRNA target site. In addition, the expression of genes upstream of the sgRNA was decreased to ~ 30% compared to their expression levels in a strain expressing a sgRNA against *gfp* (Fig. 3C).

### Inhibition of the fla/che operon increases JE1 activity

**Fig. 4:**
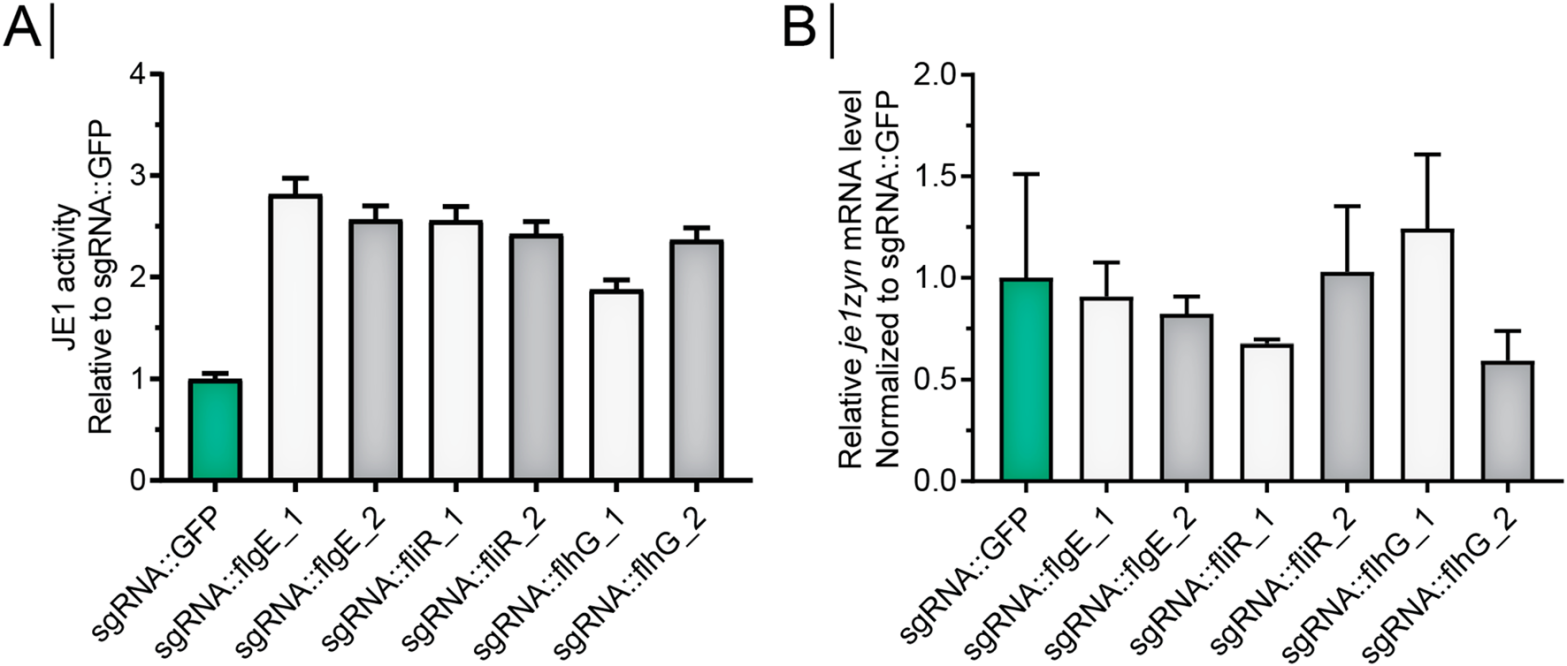
sgRNAs targeted against flagella genes increase JE1 amylase activity but not *je1zyn* mRNA level. **A** Biolector fermentation JE1 amylase activity in strains expressing sgRNA::*flgE*, sgRNA::*fliR* or, sgRNA::*flhG* (two sgRNAs per gene, grey and white bars) or sgRNA::*gfp* (green bars) normalized to sgRNA::*gfp*. N = 3, error bars depict standard deviation. **B** Relative *je1* mRNA level measured by qRT-PCR of strains as in (**A**) but error bars depict standard error of the mean

**Fig. 5:**
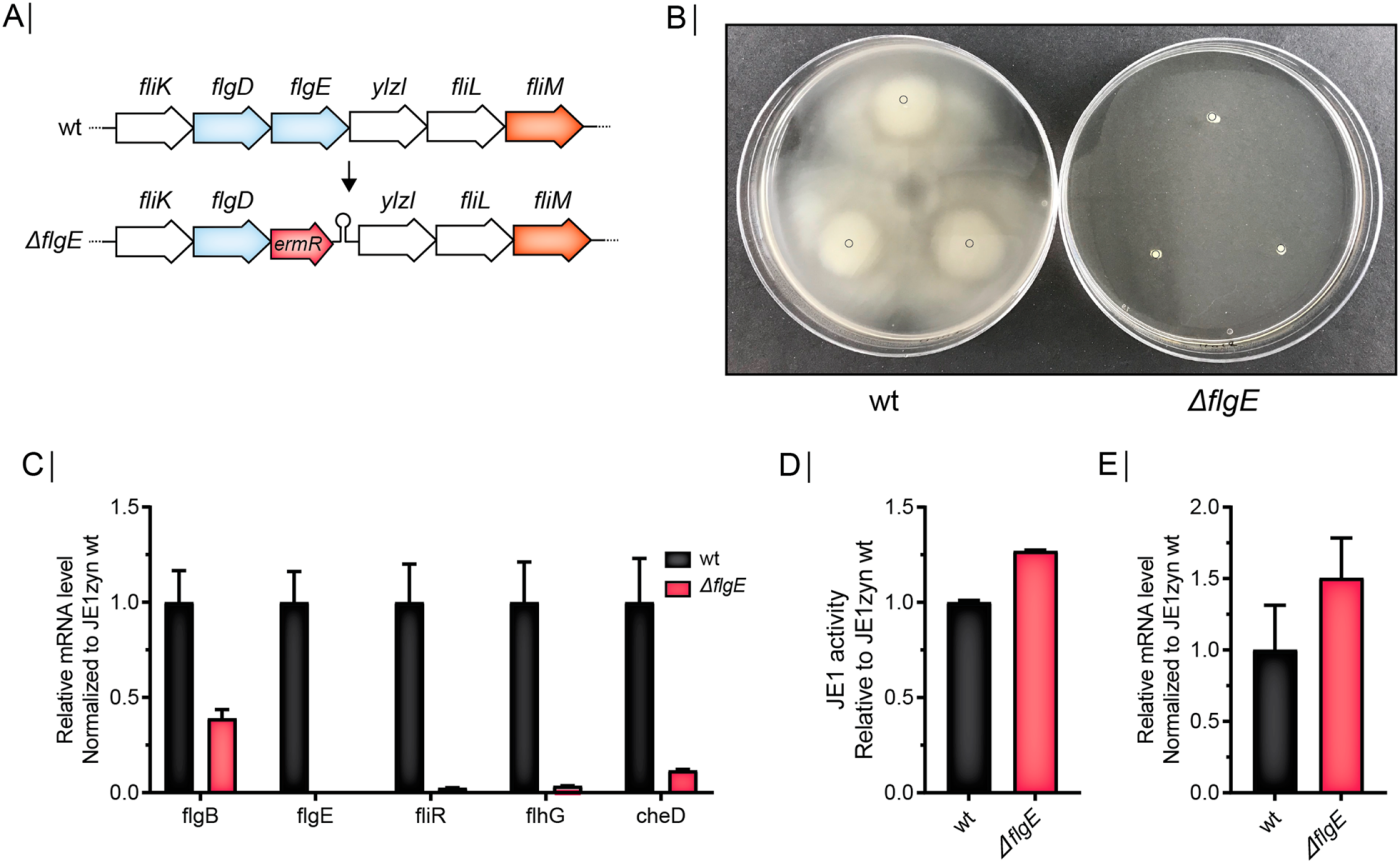
Deletion of *flgE* disrupts motility and increase JE1 yield. **A** Genomic content of the WT and Δ*flgE* strains. Deletion of *flgE* was done by replacement with an erythromycin resistance gene (ERM) followed by a terminator. **B** Swimming assay of WT and Δ*flgE* strains in triplicates. **C** Relative mRNA levels of flagella genes measured by qRT-PCR in WT (black) and Δ*flgE* (red) normalized to WT. N = 3, error bars depict standard error of the mean. **D** Biolector fermentation JE1 activity in WT (black) and Δ*flgE* (red) normalized to WT. N = 3, error bars depict standard deviation. **E** as in (**C**) but *je1zyn* mRNA levels

To examine if the CRISPR-based inhibition of the *fla/che* operon affected JE1 amylase production, we performed small-scale fermentation of the different strains and measured JE1 activity at the fermentation endpoint and also measured *je1zyn* mRNA levels from flask cultures of the strains at the late exponential growth phase. Strikingly, we found that yield measured as JE1 amylase activity increased more than 200% for all 6 strains expressing sgRNAs against *flgE, fliR* and *flhG* relative to a strain expressing a sgRNA against *gfp* (Fig. 4A). In contrast, CRISPR-based inhibition of the *fla/che* operon did not significantly affect *je1zyn* mRNA levels compared to the *gfp* reference strain, showing that the increased yield was not caused by upregulation on the mRNA level (Fig. 4B). Next, we tested if the increased yield observed for CRISPR-dCas9 repression of *flgE* can be recapitulated by replacing *flgE* with an erythromycin resistance gene followed by a transcription terminator to block polymerase transcription readthrough and mimic the dCas9 steric block. (Fig. 5A). As expected, the deletion of *flgE* together with the insertion of the terminator disrupted cell motility (Fig. 5B), abolished *flgE* expression and severely repressed expression of the downstream operon (Fig. 5C). As with the CRISPR-dCas9 targeting of *flgE,* we found that the expression of the upstream gene *flgB* was reduced (Fig. 5C). The Δ*flgE* strain showed an increase in JE1 activity of 27% compared to the wt strain (Fig. 5D). The mutant strain showed higher mRNA levels of *je1zyn*, but this was not significant (Fig. 5E).

## Discussion

In the present study, we demonstrate that inhibiting expression of the *fla/che* operon increases the α-amylase yield obtained during small-scale fermentation of a *B. subtilis* production strain. For the CRISPR-dCas9 based knockdown, we observe a 200–300% increase in yield and for the strain containing a replacement of *flgE* with an erythromycin resistance gene followed by a transcription terminator, the observed yield increase was 27%. These gains in yield are highly significant even if they can be only partly recapitulated in current *Bacillus* production strains. The *B. subtilis* strain used in this study is based on the 168 strain with a knockout of the Sigma F transcription factor to inhibit sporulation. The current production strains contain numerous genetic modifications and more work will be needed to determine how inhibition of the *fla/che* operon expression affects yields in these strains. In addition, it will be important to investigate if the findings on yield from small-scale fermentation in biolectors scales to fermentation in bioreactors.

This study was inspired by the finding that the RNA expression levels of individual genes in the *fla/che* operon are regulated during fed-batch fermentation. We found that the first half of the operon increased expression over time, while the last part of the operon was expressed at a higher level on the first day compared to later days in fermentation (Fig. 1C). This is consistent with the distance-dependent decrease of expression previously observed along the 27 kb *fla/che* operon, where overexpression of SlrA, which antagonizes SinR and SlrR, led to decreased expression of FlgE and the genes further downstream in the operon [7]. This decrease in transcript abundance along the operon is important for controlling the bistable expression of SigD as it is placed in the 3’ end of the operon [6]. Our CRISPR-dCas9 based knockdowns of the operon show clear inhibition of gene expression of genes located downstream of the sgRNAs, suggesting that the genes are predominantly expressed as a single transcriptional unit. However, we detect a low but significant expression of CheD when transcription is blocked by the recruitment of sgRNAs or the inserted terminator upstream in the operon. This was surprising as the only promoter annotated downstream to FlgE is one immediately upstream to SigD [1, 38], which would not transcribe CheD as it is placed upstream to the promoter. It is possible that the low but significant expression of CheD in our knockdown and deletion strains is due to exo- and endonucleases targeting only upstream parts of the operon mRNA for degradation or that a low activity and hitherto unknown promoter becomes activated. Nevertheless, our finding that inhibition of the *fla/che* operon increases the α-amylase yield suggests that important regulation is taking place at this operon during fermentation and that at least a subset of cells obtain a motile phenotype during fermentation.

We demonstrate that both CRISPR-dCas9 based knockdown of the *fla/che* operon and replacement of *flgE* with an erythromycin resistance gene followed by a transcription terminator leads to significant increase in yield, but the mechanism responsible remains unclear. The large flagella structure is expensive to build and in addition requires a lot of energy to operate, meaning that its inhibition will preserve energy that could be used for protein synthesis instead. However, the fact that CRISPR-dCas9 based knockdown shows a much higher yield than the deletion of *flgE* followed by a transcription terminator indicates that the mechanism depends on broader regulatory changes occurring in the cells. Especially because the effect of the two types of *fla/che* operon inhibition on the expression of the genes in the operon is similar (Fig. 3C and Fig. 5C). It is known that the activation of the *fla/che* operon expression is central to the decision whether to enter the motile phenotype [16]. The operon is expressed from a main promoter controlled by SigA and DegU/SwrA and a secondary upstream promoter controlled by SigD. Phosphorylated DegU binds to the main promoter and represses the expression of the operon. In non-domesticated strains, the binding of SwrA to the phosphorylated DegU will lead to induction of the operon, leading to the expression of SigD, which in turn can activate the *sigD* dependent promoter, serving as an ON/OFF switch ensuring robust expression of the *fla/che* operon and additional SigD dependent genes needed for flagella assembly and operation [26]. In this way, SwrA/SigD sets the threshold for cells to commit to swimming [16]. However, in lab strains, such as the one used here, SwrA is inactivated, which will reduce the tendency of cells to become motile and make it impossible for cells to acquire enough flagella to swarm over surfaces. Mutants expressing DegU32(hy), which shows increased levels of phosphorylated DegU deficient for binding to SwrA, will display the Hy phenotype characterized by no motility and hypersecretion of digestive enzymes, and it is possible that our inhibition of the *fla/che* operon results in some of the same regulatory changes observed in the Hy mutants, possibly driven by other regulatory targets of phosphorylated DegU. In our experiments, the transcriptional blockage by CRISPR-dCas9 recruitment or the insertion of a transcription terminator will be expected to further alleviate the possibility for positive feedback acting on *sigD* expression. This is consistent with our observation that also genes upstream of the transcriptional block show reduced expression (Fig. 3C and Fig. 5C).

It is puzzling, that we observe a more prominent increase in yield using CRISPR-dCas9 based knockdown of the *fla/che* operon than for the replacement of *flgE*, with an erythromycin resistance gene followed by a transcription terminator. Although the regulatory effect of these two types of inhibition seems quite similar (Fig. 3C and Fig. 5C), there are differences in the set-up, which could explain the observed differences in yield. Notably, the mRNA levels were measured in shake flask cultures, meaning that other types of regulation could occur during fermentation. Alternatively, the difference could be caused by the knockout of *flgE,* which will completely inhibit flagella assembly. It is possible that the partly assembled flagella lacking *flgE,* which is the component that links the basal body to the hook, could cause downstream effects that inhibit yield. One such effect could be the secretion of the anti-SigD factor FlgM [4], which is dependent on hook-basal body completion for secretion, meaning that the lack of FlgE could influence the threshold of *sigD* activation [3].

## Conclusion

In conclusion, we demonstrate that the inhibition of genes in the *fla/che* operon in *B. subtilis* increases the yield of amylase production and that creation of CRISPRi strains with sgRNAs targeting flagella, and potentially other genes, is a promising strategy for optimizing fermentation yield. Interestingly, CRISPRi mediated repression of the *fla/che* operon results in higher yields than knockout of *flgE* and insertion of a transcriptional terminator. This suggests that the CRISPRi mediated yield improvement occurs by a complex mechanism, which is favored by reduced flagella production rather complete inhibition.

## Methods

### Media

*Bacillus* strains were grown on LB agar (10 g/L Tryptone, 5 g/L yeast extract, 5 g/L NaCI, 15 g/L agar) plates or in TY liquid medium (20 g/L Tryptone, 5 g/L yeast extract, 7 mg/L FeCI_2_, 1 mg/L MnCI_2_, 15 mg/L MgCI_2_) or YT medium (8 g/L Tryptone, 5 g/L yeast extract, 5 g/L NaCl) supplemented with 5 or 1 µg/ml erythromycin, 6 µg/ml chloramphenicol, 120 µg/mL spectinomycin, or 0.4 mg/mL D-alanine when appropriate. Transformation of Bacilli was done in Spizizen I medium (6 g/L KH_2_PO_4_, 14 g/L K_2_HPO_4_, 2 g/L (NH4)_2_SO_4_, 1 g/L sodium citrate, 0.2 g/L MgSO_4_ pH 7.0, 0.5% glucose, 0.1% yeast extract and 0.02% casein hydrolysate).

### Cloning

Competent cells and transformation of *B. subtilis* was obtained as described in [39]. Genomic DNA was prepared by using the commercially available QIAamp DNA Blood Kit from Qiagen. The respective DNA fragments were amplified by PCR using the Phusion Hot Start DNA Polymerase system (Thermo Scientific). PCR amplification reaction mixtures contained 1 µL (0.1 pg) of template DNA, 1 µL of sense primer (20 pmol/µL), 1 µL of anti-sense primer (20 pmol/µL), 10 µL of 5X PCR buffer with 7,5 mM MgCl_2_, 8 µL of dNTP mix (1.25 mM each), 39 µL water, and 0.5 µL (2 U/) DNA polymerase.

The condition for SOE-PCR [14] is as follows: purified PCR products were used in a subsequent PCR reaction to create a single fragment using splice overlapping PCR (SOE) using the Phusion Hot Start DNA Polymerase system (Thermo Scientific) as follows. Primers complementary to the very 3'-end of each strand of the outer PCR products were added and a thermocycler was used to assemble and amplify the SOE fragment.

### Construction of B. subtilis strains containing heterologous genes

The base *B. subtilis* strain is built on strain 168 [18] with the following deletions: *sigF, nprE, aprE, amyE, and srfAC*. The deletions renders them all inactive as described in Sloma and Christianson [35]. The α-amylase JE1 was obtained from *B. halmapalus* (aka *Sutcliffiella halmapala*) and later codon-optimized for *B. subtilis* into JE1zyn with a Novozymes proprietary codon optimization model. In brief, a synthetic gene for *JE1zyn*, driven by the P4199 promoter [15], was ordered from GeneArt and fused to a chloramphenicol resistance marker by SOE PCR and integrated into the *PEL* locus by homologous recombination, yielding ThKK0007. The JE1zyn expression cassette (without flanks) is Sequence 1 in Additional File 3. As in Geissler et al. [12], the C*as9d* gene cassette [33] under control of the P4199 promoter, was likewise ordered from GeneArt. The *Cas9d* cassette, fused to a spectinomycin resistance marker, was integrated into the *AmyE* locus, yielding ThKK0016. The Cas9d expression cassette (without flanks) is Sequence 2 in Additional File 3. The *flgE* knockout was made by fusing the *Erm* gene including its natural terminator from pE194 with the up- and downstream flanking region of *flgE* by SOE-PCR. The flanking region were designed so only the open reading frame of *flgE* would be substituted by the *Erm* resistance marker. The SOE fragment was transformed into ThKK0007 and transformants were selected of 1 µg/mL ERM LB plates. The resulting strain was BT11018.

### sgRNA cloning

The sgRNA cloning was performed as described in Geissler et al. [12]. In brief, the sgRNA was expressed from a Pq promoter and the initial expression cassette was ordered from GeneArt as a DNA sequence with a sgRNA spacer sequence directed towards GFP (sequence 3 in Additional File 3). The sgRNA expression construct was integrated into the *alr* locus, yielding ThKK0086. The downstream flanking region of the sgRNA expression carries the wild type *alr* sequence, which upon homologous recombination, cures the D-alanine auxotrophy and enables the strain to grow without additional D-alanine supplementation. Thus, the transformants were selected on LB plates. Due to the D-alanine auxotrophy of ThKK0086, 400 µL 10 mg/mL D-alanine was added to 10 ml Spitz transformation media for transformation. The strains harboring the sgRNA::*gfp* was named ThKK0108.

The 20 bp spacer sequence was substituted to a new spacer sequence by PCR amplifying the upstream *alr* flanking region plus the Pq promoter of the sgRNA expression cassette in ThKK0108. The reverse primer for this reaction carried the new 20 bp spacer sequence as an overhang. A second PCR was made by amplifying the sgRNA scaffold, directly downstream of the spacer sequence, plus the downstream *alr* flanking region from ThKK0108. The forward primer in the reaction carried the new spacer sequence in an overhang. The two fragments were combined in an SOE-PCR and transformed into ThKK0086 and selected for growth on LB plates w/o additional D-alanine addition. Full list of spacer sequences is found in (Additional File 1: Table S3).

### Fermentations

Standard lab fermentations were performed at 38 °C, with a pH of 6.8 – 7.2 (regulated with NH_4_OH and H_3_PO_4_, respectively), an aeration of 1.5 l/min/kg broth weight and an agitation of 1500 rpm. The feed strategy started with a 0.05 g/min/kg initial broth after inoculation (0 h) and shifted to 0.156 g/min/kg initial broth after inoculation until the end. The cultivation was run for five days with constant agitation, and the oxygen tension was measured with a dissolved oxygen electrode and followed online in this period. OD_650_ measurements were performed to monitor culture density. Fermentation was run in a non-commercial 5L bioreactor built by Novozymes A/S on site.

#### Small-scale fermentation

Small-scale fermentations were performed in the Biolector II (m2p-labs). Strains were fermented in flower plates (MTP-48-B) in 1 mL TY media for 24 h at 37 °C, 1000 rpm in the Biolector (m2p-labs). The cultivation plates were inoculated from an overnight culture grown in 10 mL TY media at 37 ˚C, 250 rpm. The flower plates were inoculated to OD_450_ 0.05.

### Amylase assay

Amylase activity was measured in culture supernatants using the AMYL (Roche/Hitachi # 11,876,473 001). Culture supernatants from the Biolector were diluted to 1/50 in Stabilizer buffer (0.03 M CaCl2; 0.0083% Brij 35). Supernatants from bioreactor samples were diluted ~ 1/6000 in Stabilizer buffer. 20 µL diluted sample was mixed with 180 µL assay substrate, consisting of a 1:10 dilution of solution 2 into solution 1 provided by the kit, and then incubated at 37 °C for 30 min w/shaking at 700 rpm. Absorbance was measured at 405 nm in a plate reader. An in-house JE1 standard was included from the final activity value, KNU(N)/g, was determined.

### Swimming assay

Strains were inoculated into 10 mL TY ON at 37 °C 250 rpm. The overnight cultures were diluted to OD_450_ 0.05 in 10 mL and grown for 4 h at 37 °C 250 rpm. 2 µL culture was spotted onto a freshly prepared 0.26% LB-agar plate by pouring 15 mL liquid LB-agar into a petri-dish and dried for 10 min before spotting. After spotting, plates were dried for additional 5 min before placing at 37 °C overnight.

### RNA extractions

Fermentation samples were harvested by mixing cell culture with 100% ethanol in 1:1 volume, immediately storing on dry ice before transferring to − 80 °C. Samples for qRT-PCR were obtained from overnight cultures in YT medium of each strain in triplicates diluted to OD_450_ 0.05 before harvesting at OD_450_ ~ 0.8. For all samples, cells were collected at 3,220 g for 4 min at 4 °C. Pellets were vortexed in 0.5 ml glass beads (Sigma #G8772), 1 ml extraction buffer (10 mM NaOAc, 150 mM sucrose, 1% SDS), and 1 ml phenol:chloroform 5:1 pH 4.5 (ThermoFisher #AM9720) for 4 min and glass beads were removed. Samples were incubated for 5 min at 65 °C before freezing in liquid nitrogen and centrifuged at 13,000*g* for 20 min at 4 °C before transferring the aqueous phase to repeat the hot phenol extraction. The aqueous phase was then transferred to 1 volume of chloroform and inverted before centrifugation at 13,000*g* for 10 min at 4 °C for phase separation. RNA was finally precipitated in 1 volume of isopropanol at room temperature for 10 min before centrifugation at 15000*g* for 45 min at 4 °C. RNA pellets were washed with 70% ethanol and dissolved in water. DNase digestion was performed for qRT-PCR samples using TURBO DNase (Invitrogen #AM2238) and purified using RNA Clean & Concentrator (Zymo research #R1016) according to the manufacturer’s instructions. DNase digestion was performed for fermentation RNA-seq samples using DNase I (Qiagen #79,254) and purified using RNeasy MinElute Cleanup Kit (Qiagen #74,204) according to the manufacturer’s instructions. RNA integrity was assessed using gel electrophoresis or bioanalyzer.

### qRT-PCR

Quantitative RT-PCR was performed using Brilliant III Ultra-Fast SYBR Green qRT-PCR Master Mix (Agilent Technologies #600,886) according to the manufacturer’s protocol with 5 ng RNA in 10 µL reactions using 0.5 µM of each primer (Additional File 1: Table S1). Each of three biological replicates was quantified in technical duplicates using Quantstudio 6 Flex (Applied Biosystems #4,485,694) incubating at 50˚C for 10 min, 95 °C for 3 min and 40 cycles of 95 °C for 5 s and 60 °C for 15 s. Fold changes were calculated using the 2^−ΔΔCt^ method and *citA* was used as a reference gene.

### Transcriptome analysis

The transcriptome analysis from Geissler et al. [11] used here, was based on RNA-seq data deposited in GEO (GSE189556). In summary, samples were sequenced by BGI using the DNBSEQ™ platform. The RNA-seq data was quality-processed, mapped, and expression quantified as in Geissler et al. [12]. The gene information is as annotated in the BSGatlas v1 [10]. The transcriptome was analyzed for differential expression following the procedure in Geissler et al. [11] and make use of the nine pair-wise tests (Additional File 1: Figure S1) with DESeq2 (version 1.22.1) [22] in R (version 3.5.1) [31]. Differentially expressed genes were selected according to FDR adjusted p ≤ 0.05.

## Supplementary Information

**Additional file 1**: **Figure S1.** Overview of pairwise tests.** Figure S2.** Differentially expressed gene abundances. **Table S1.** qRT-PCR primers. **Table S2.** Genotypes of strains used in this study. **Table S3.** List of spacer sequences and their target genes.

**Additional file 2**: Expression data for the genes in the Flagella operon.

**Additional file 3:** Expression cassette sequences.

## Data Availability

The dataset used to obtain the data presented in Fig. 1 is available in the GEO repository as GSE189556. The specific subset of the data supporting the conclusions of this article is available as Additional file 2.
