# Supplementary material for: Flagella disruption in Bacillus subtilis increases amylase production yield
Source: Microb Cell Fact. 2022 Jul 2;21:131. doi: 10.1186/s12934-022-01861-x (PMC9250202; doi:10.1186/s12934-022-01861-x)
Supplement: Supplementary file 1 — Additional file 1: Figure S1. Overview of pairwise tests. Figure S2. Differentially expressed gene abundances. Table S1. qRT-PCR primers. Table S2. Genotypes of strains used in this study. Table S3. List of spacer sequences and their target genes. [file 12934_2022_1861_MOESM1_ESM.pdf]

Additional file 1 for:

Flagella disruption in *Bacillus subtilis* increase amylase production yield

Annaleigh Ohrt Fehler, Thomas Beuchert Kallehauge, Adrian Sven Geissler, Enrique González-Tortuero, Stefan Ernst Seemann, Jan Gorodkin, Jeppe Vinther

Content:

Supplementary Figure 1: Overview of pairwise tests.

Supplementary Figure 2: Differentially expressed gene abundances.

Supplementary table 1: qRT-PCR primers

Supplementary table 2: Genotypes of strains used in this study.

Supplementary table 3: List of spacer sequences and their target genes.

Supplementary Figure 1:

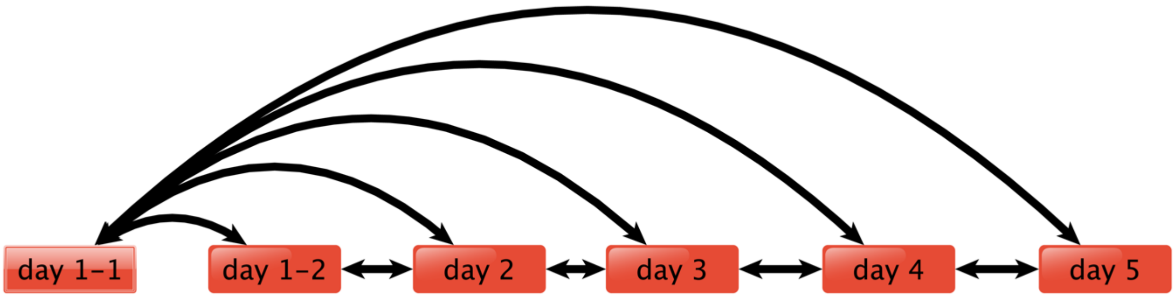

Supplementary Figure 1: Overview of pairwise tests. Pair-wise tests (arrows) for differential expression that were conducted between the RNA-seq data that were taken at 6 time-points (red boxes).

Supplementary Figure 2:

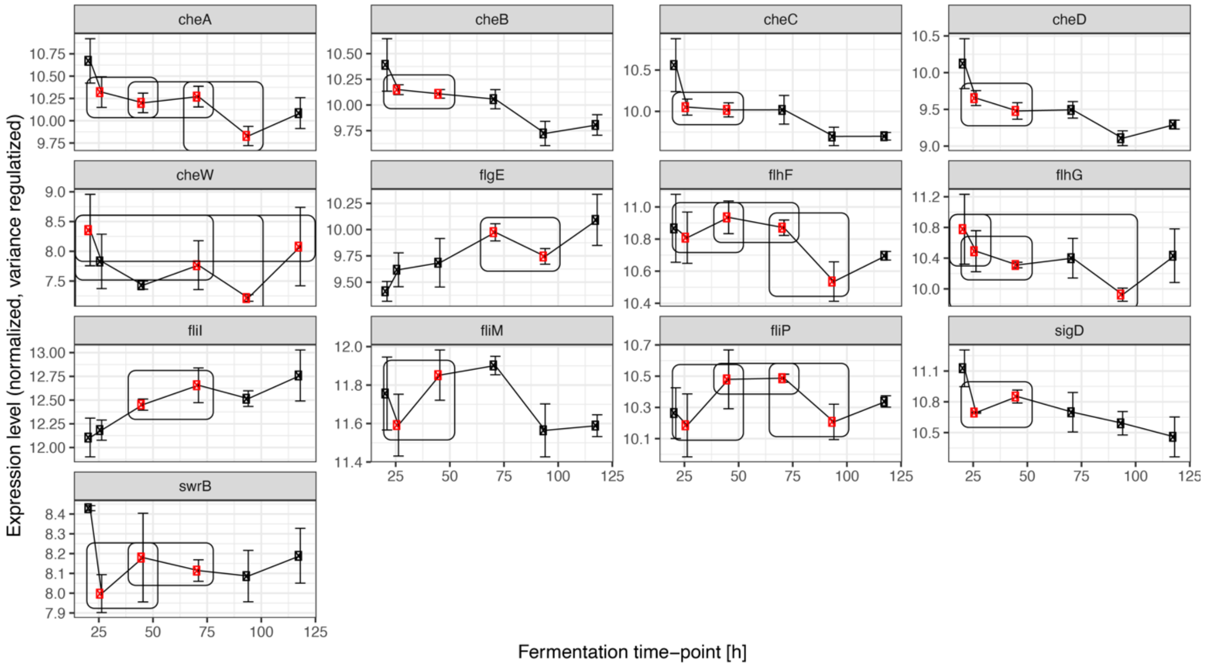

Supplementary Figure 2: Differentially expressed gene abundances.

Differentially expressed gene abundances. Shown are the temporal expression dynamics from the RNA-Seq data for the 13 genes of the *fla/che* operon that had statistically significant changes in expression levels during the fermentation according to a test with DESeq2 (FDR adjusted $p \leq 0.05$, 5 comparisons along time axis and 4 against expression at first time-point). The relative expression levels shown here were size-factor normalized and rlog transformed. In contrast to the heatmap of Figure 1C, the data was not z-scaled per gene. Rounded boxes and red highlights indicate which comparison pair (Supplementary Figure 1) was significant.

Supplementary Table 1: qRT-PCR primers

Target	Sequence	fwd/rev
JE1zyn	TGGGACGATGATGCAGTATTT	fwd
JE1zyn	TGGTATCCAGATCGCAGTTATG	rev
citA	GCCAAACAACATGGACGATATG	fwd
citA	GCAATGATAGAAGGCGTGATTG	rev
flgE	TCGGCGGAACAAACTCTAAG	fwd
flgE	GTCGCCGTCAATTGCTAAATC	rev
flgB	CCTTGAGCAGAGCGGATATT	fwd
flgB	GACGCGAGGATTCTTGATCT	rev
fliR	CGGCCTATGTCTGGGTTTAAT	fwd
fliR	CCGGTTTGCGGATCAATAAC	rev
fliQ	GATGATCAGCGGGCCATTAT	fwd
fliQ	TAAACGCCAAAGTCTGTTCCT	rev
flhG	GCGTTCTGCCAATGGTTTATTC	fwd
flhG	CAGCGCAAGTGCCATATTTAAG	rev
CheD	GTCTTGTTCATGTCATGCTTCC	fwd
CheD	AGCATATCAATCGTGGTCTGT	rev

Supplementary Table 2: Genotypes of strains used in this study. All strains are in *Bacillus subtilis 168* background.

**Strain**	**Name**	**Genotype**
ThKK0012		*ΔspoIIAC, ΔamyE, Δapr, ΔnprE, ΔsrfAC*
ThKK0007	JE1zyn	*ΔspoIIAC, ΔamyE, Δapr, ΔnprE, ΔsrfAC, pel::P4199-JE1zyn-Cat*
ThKK0016	JE1zyn + dCas9	*ΔspoIIAC, ΔamyE, Δapr, ΔnprE, ΔsrfAC, pel::P4199-JE1zyn-Cat, amyE::P4199-dCas9*
ThKK0086		*ΔspoIIAC, ΔamyE, Δapr, ΔnprE, ΔsrfAC, pel::P4199-JE1zyn-Cat, amyE::P4199-dCas9, alr-::Neo,*
ThKK0108	sgRNA::*gfp*	*ΔspoIIAC, ΔamyE, Δapr, ΔnprE, ΔsrfAC, pel::P4199-JE1zyn-Cat, amyE::Spec P4199-dCas9, alr+::Ban-sgRNA::gfp*
ThKK0089	sgRNA::*JE1zyn*	*ΔspoIIAC, ΔamyE, Δapr, ΔnprE, ΔsrfAC, pel::P4199-JE1zyn-Cat, amyE::Spec P4199-dCas9, alr+::Ban-sgRNA::JE1zyn*
ThKK0273	sgRNA::flgE_1	*ΔspoIIAC, ΔamyE, Δapr, ΔnprE, ΔsrfAC, pel::P4199-JE1zyn-Cat, amyE::Spec P4199-dCas9, alr+::Ban-sgRNA::sgRNA_19_003_1*
ThKK0274	sgRNA::flgE_2	*ΔspoIIAC, ΔamyE, Δapr, ΔnprE, ΔsrfAC, pel::P4199-JE1zyn-Cat, amyE::Spec P4199-dCas9, alr+::Ban-sgRNA::sgRNA_19_003_2*
ThKK0275	sgRNA::fliR_1	*ΔspoIIAC, ΔamyE, Δapr, ΔnprE, ΔsrfAC, pel::P4199-JE1zyn-Cat, amyE::Spec P4199-dCas9, alr+::Ban-sgRNA::sgRNA_19_003_3*
ThKK0276	sgRNA::fliR_2	*ΔspoIIAC, ΔamyE, Δapr, ΔnprE, ΔsrfAC, pel::P4199-JE1zyn-Cat, amyE::Spec P4199-dCas9, alr+::Ban-sgRNA::sgRNA_19_003_4*
ThKK0277	sgRNA::flhG_1	*ΔspoIIAC, ΔamyE, Δapr, ΔnprE, ΔsrfAC, pel::P4199-JE1zyn-Cat, amyE::Spec P4199-dCas9, alr+::Ban-sgRNA::sgRNA_19_003_5*
ThKK0278	sgRNA::flhG_2	*ΔspoIIAC, ΔamyE, Δapr, ΔnprE, ΔsrfAC, pel::P4199-JE1zyn-Cat, amyE::Spec P4199-dCas9, alr+::Ban-sgRNA::sgRNA_19_003_6*
BT11018	*ΔflgE*	*ΔspoIIAC, ΔamyE, Δapr, ΔnprE, ΔsrfAC, pel::P4199-JE1zyn-Cat, ΔflgE::Erm*

Supplementary Table 3: List of spacer sequences and their target genes.

Target gene	Gene ID	Reference	sgRNA name	spacer sequence	Strain
GFP			sgRNA::GFP	TCTGTTAGTGGAGAGGGTGA	ThKK0108
JE1zyn			sgRNA::JE1zyn	GAATCATGAAACAACAAAAA	ThKK0089
flgE	936497	NC_000964.3	sgRNA_19_003_1	TTTATCCAATCCATCACTGT	ThKK0273
flgE	936497	NC_000964.3	sgRNA_19_003_2	TAGCATTGGTTCTGATGGAA	ThKK0274
fliR	940130	NC_000964.3	sgRNA_19_003_3	AAACCTATGCGATGAACAGC	ThKK0275
fliR	940130	NC_000964.3	sgRNA_19_003_4	ACATTTCGATGTCCAAATAG	ThKK0276
flhG	940123	NC_000964.3	sgRNA_19_003_5	TCTTCTGCTGATAAAATAAA	ThKK0277
flhG	940123	NC_000964.3	sgRNA_19_003_6	CAAACAGCACATAATCGAAC	ThKK0278
