# Supplementary material for: Flagella disruption in Bacillus subtilis increases amylase production yield
Source: Microb Cell Fact. 2022 Jul 2;21:131. doi: 10.1186/s12934-022-01861-x (PMC9250202; doi:10.1186/s12934-022-01861-x)
Supplement: Supplementary file 3 — Additional file 3: Expression cassette sequences. [file 12934_2022_1861_MOESM3_ESM.docx]

>Sequence #1 – JE1zyn expression cassette

TTGTTAAAAATTCGGAATATTTATACAATATCATATGTATCACATTGAAAGGGAGGAGAATCATGAAACAACAAAAACGGCTTTACGCCCGATTGCTGACGCTGTTATTTGCGCTCATCTTCTTGCTGCCTCATTCTGCAGCCGCGGCACATCACAATGGTACGAATGGGACGATGATGCAGTATTTCGAGTGGCATCTGCCGAATGACGGCAACCATTGGAACAGGCTGCGGGACGACGCGTCCAATTTAAGGAATCGCGGCATAACTGCGATCTGGATACCACCGGCGTGGAAGGGTACGTCACAAAACGACGTGGGATATGGAGCGTACGACCTCTACGATTTAGGAGAATTCAACCAGAAAGGCACAGTTCGAACAAAGTACGGGACGCGCTCTCAATTGGAGTCAGCGATCCATGCTCTGAAAAACAACGGCGTTCAAGTTTATGGCGATGTTGTCATGAACCATAAAGGCGGTGCAGATGCGACAGAAAATGTGCTCGCAGTTGAAGTAAATCCAAACAATCGAAACCAAGAGATTTCCGGAGACTATACGATCGAAGCCTGGACAAAATTTGATTTTCCAGGTCGGGGCAACACATATAGTGATTTTAAATGGCGCTGGTACCATTTCGACGGCGTAGATTGGGATCAGTCACGGCAATTCCAAAACCGAATCTATAAGTTTAGAGGCAAAGCTTGGGACTGGGAAGTTGATTCGGAAAACGGAAACTATGATTATTTAATGTATGCGGACGTGGATATGGATCATCCGGAGGTGGTGAACGAACTTCGCCGTTGGGGGGAATGGTATACCAATACGTTAAACCTGGATGGATTTCGCATAGATGCCGTGAAACATATTAAATACTCATTTACCCGGGATTGGTTAACGCATGTCCGGAACGCCACGGGTAAAGAGATGTTCGCCGTCGCGGAATTTTGGAAAAATGACCTGGGCGCCTTGGAAAATTACCTTAACAAAACGAACTGGAATCACAGCGTCTTTGACGTACCGCTTCACTACAACTTATATAATGCATCTAACTCAGGAGGCAATTATGACATGGCCAAGCTTTTGAATGGAACGGTGGTTCAAAAGCACCCGATGCATGCAGTGACGTTCGTCGATAACCATGATTCACAGCCTGGGGAGTCCCTCGAAAGTTTCGTCCAGGAGTGGTTCAAACCATTAGCATATGCCCTTATTTTGACGAGGGAACAAGGATATCCTAGTGTTTTTTACGGCGACTATTATGGAATCCCGACACATTCTGTGCCGGCCATGAAGGCAAAAATCGATCCAATCTTGGAAGCGCGTCAAAACTTCGCCTATGGGACGCAACATGATTACTTTGACCACCATAATATTATTGGATGGACACGCGAAGGGAATACCACACACCCCAATTCAGGATTAGCAACAATTATGTCGGACGGTCCAGGGGGCGAAAAATGGATGTATGTCGGACAAAACAAAGCAGGCCAAGTGTGGCATGACATAACAGGCAATAAACCGGGGACAGTGACGATTAACGCAGATGGCTGGGCAAATTTTTCAGTCAACGGAGGTTCGGTCTCCATTTGGGTGAAAAGATAAATCAATAAAAAAACGCTGTGCGGTTAAAGGGCACAGCGTTTTTTTTGTGTAT

>Sequence #2 – Cas9d expression cassette

GCAACGTTCGCAGATGCTGCTGAAGAGATTATTAAAAAGCTGAAAGCAAAAGGCTATCAATTGGTAACTGTATCTCAGCTTGAAGAAGTGAAGAAGCAGAGAGGCTATTGAATAAATGAGTAGAAAGCGCCATATCGGCGCTTTTCTTTTGGAAGAAAATATAGGGAAAATGGTACTTGTTAAAAATTCGAAATATTTATACAATATCATATGTATCACATTGAAAGGGGAGGAGAATCATGGACAAAAAATACAGCATCGGCCTGGCTATTGGCACAAATTCAGTTGGCTGGGCAGTTATCACAGACGAATATAAAGTTCCGAGCAAAAAATTTAAAGTCCTGGGCAATACAGATCGCCATAGCATCAAAAAAAACCTGATTGGCGCACTGCTGTTTGATTCAGGCGAAACAGCAGAAGCAACAAGACTTAAAAGAACAGCAAGACGCAGATATACAAGACGCAAAAATCGCATTTGCTATCTGCAAGAAATCTTTAGCAACGAAATGGCGAAAGTCGACGACAGCTTTTTTCATAGACTGGAAGAATCATTTCTGGTCGAAGAAGATAAAAAACACGAACGCCATCCGATTTTTGGCAACATTGTTGATGAAGTCGCGTATCATGAAAAATACCCGACAATTTATCATCTGCGCAAAAAACTGGTTGACAGCACAGATAAAGCAGATCTTCGCCTGATTTATCTGGCACTGGCACATATGATCAAATTTAGAGGCCATTTTCTGATCGAAGGCGATCTGAATCCGGATAATTCAGATGTCGACAAACTGTTTATTCAGCTGGTCCAGACATATAACCAGCTGTTTGAAGAAAATCCGATTAATGCATCAGGCGTTGATGCAAAAGCAATTCTGTCAGCAAGACTGTCAAAATCAAGACGCCTGGAAAATCTGATTGCACAACTGCCTGGCGAAAAAAAAAATGGACTGTTTGGCAATCTTATTGCACTGTCACTGGGCCTGACACCGAACTTTAAATCAAATTTTGATCTGGCGGAAGATGCGAAACTGCAACTTTCAAAAGATACGTATGATGACGATCTGGATAATCTGCTGGCGCAAATTGGCGATCAATATGCAGATCTTTTTCTGGCAGCGAAAAATCTGTCAGATGCAATTCTGCTGTCAGATATTCTGCGCGTCAATACAGAAATTACAAAAGCACCGCTGAGCGCGAGCATGATTAAAAGATATGATGAACATCATCAGGACCTGACACTGCTGAAAGCACTGGTTAGACAACAACTGCCGGAAAAATACAAAGAAATCTTTTTTGATCAGAGCAAAAACGGCTATGCAGGCTATATTGATGGCGGAGCATCACAAGAAGAATTTTACAAATTTATCAAACCGATCCTGGAAAAAATGGATGGAACAGAAGAACTGCTGGTTAAACTGAATCGCGAAGATTTACTGAGAAAACAGCGCACATTTGATAATGGCTCAATTCCGCATCAAATTCATCTGGGCGAACTGCATGCGATTCTTAGACGCCAAGAAGATTTTTATCCGTTTCTGAAAGACAACCGGGAAAAAATTGAAAAAATCCTGACATTTCGCATCCCGTATTATGTCGGACCGCTGGCAAGAGGCAATTCAAGATTTGCATGGATGACACGCAAAAGCGAAGAAACAATTACACCGTGGAATTTTGAAGAAGTCGTTGATAAAGGCGCAAGCGCACAATCATTTATTGAACGCATGACGAACTTTGACAAAAACCTGCCGAATGAAAAAGTCCTGCCGAAACATTCACTGCTGTATGAATACTTTACGGTCTATAATGAACTGACGAAAGTCAAATATGTCACAGAAGGCATGAGAAAACCGGCATTTCTGTCAGGCGAACAGAAAAAAGCGATTGTCGATCTTCTGTTTAAAACGAACCGCAAAGTCACAGTGAAACAGCTGAAAGAAGATTACTTTAAAAAAATCGAATGCTTTGATAGCGTCGAAATCTCAGGCGTCGAAGATAGATTTAATGCAAGCCTGGGCACATATCATGATCTGCTGAAAATCATCAAAGATAAAGATTTTCTGGATAACGAAGAAAACGAAGATATCCTGGAAGATATTGTGCTGACACTGACGCTTTTTGAAGATCGCGAAATGATTGAAGAACGCCTGAAAACATATGCGCACCTGTTTGATGATAAAGTCATGAAACAACTTAAACGCAGACGCTATACAGGCTGGGGCAGACTTTCAAGAAAACTGATTAACGGCATTCGCGATAAACAAAGCGGCAAAACAATCCTGGATTTTCTGAAATCAGATGGCTTTGCGAATCGCAATTTTATGCAGCTGATTCATGATGACAGCCTGACGTTTAAAGAAGATATTCAGAAAGCACAAGTTTCAGGCCAAGGCGATTCACTGCATGAACATATTGCAAATCTGGCAGGCTCACCGGCAATCAAAAAAGGCATTCTGCAAACAGTTAAAGTCGTCGATGAACTGGTTAAAGTTATGGGCAGACATAAACCGGAAAACATCGTTATTGAAATGGCACGCGAAAATCAGACAACACAAAAAGGACAGAAAAATTCACGCGAACGGATGAAAAGAATTGAAGAAGGCATTAAAGAACTGGGCAGCCAAATCCTGAAAGAACATCCGGTTGAAAATACACAGCTGCAGAACGAAAAACTGTATCTGTATTATCTGCAGAATGGACGCGATATGTATGTCGATCAAGAACTGGATATTAATCGCCTGAGCGATTATGATGTGGATGCTATTGTTCCGCAGAGCTTTCTTAAAGATGATAGCATCGATAACAAAGTCCTGACACGCTCAGATAAAAACAGAGGCAAATCAGATAATGTCCCGTCAGAAGAGGTTGTCAAAAAAATGAAAAACTACTGGCGTCAACTGCTGAACGCGAAACTTATTACACAACGCAAATTTGACAATCTGACAAAAGCAGAAAGAGGCGGACTGTCAGAACTTGATAAAGCGGGTTTTATCAAAAGACAGCTGGTCGAAACACGCCAGATTACAAAACATGTTGCGCAAATTCTGGATAGCCGCATGAACACAAAATATGACGAAAACGATAAACTGATCCGGGAAGTCAAAGTCATTACGCTGAAATCAAAACTGGTCAGCGATTTTCGCAAAGACTTTCAGTTTTACAAAGTCCGCGAAATCAACAACTACCATCATGCACATGATGCATATCTGAATGCAGTTGTCGGCACAGCGCTTATCAAAAAATACCCTAAACTGGAAAGCGAATTTGTCTACGGCGACTATAAAGTCTATGATGTCCGCAAAATGATTGCGAAAAGCGAACAAGAAATTGGCAAAGCGACAGCGAAATACTTTTTTTACAGCAACATCATGAACTTTTTTAAAACGGAAATCACACTGGCGAACGGCGAAATTAGAAAAAGACCGCTTATTGAAACGAACGGTGAAACAGGCGAAATTGTTTGGGATAAAGGCAGAGATTTTGCAACAGTTAGAAAAGTTCTGAGCATGCCGCAAGTCAACATCGTGAAAAAAACAGAAGTTCAGACAGGCGGATTTAGCAAAGAATCAATTCTTCCGAAACGCAACTCAGACAAACTGATTGCGCGTAAAAAAGACTGGGACCCGAAAAAATACGGTGGCTTTGATTCACCGACAGTTGCATATTCAGTTCTGGTTGTTGCGAAAGTGGAAAAAGGCAAATCCAAAAAACTTAAAAGCGTGAAAGAACTTCTGGGCATCACAATTATGGAACGCTCGAGCTTTGAAAAAAACCCGATCGACTTTCTGGAAGCCAAAGGCTATAAAGAAGTGAAAAAAGACCTTATTATCAAACTGCCGAAATACAGCCTGTTTGAACTGGAAAATGGCAGAAAACGCATGCTGGCATCAGCAGGCGAACTTCAGAAAGGCAATGAACTGGCACTGCCGTCAAAATATGTTAACTTTCTGTATCTGGCGAGCCATTACGAAAAACTTAAAGGCTCACCGGAAGATAACGAACAGAAACAACTGTTTGTCGAACAGCATAAACATTACCTGGACGAAATCATCGAACAAATCAGCGAATTTTCAAAACGCGTTATTCTGGCAGATGCGAACCTGGATAAAGTTCTTAGCGCATATAACAAACACCGGGATAAACCGATTAGAGAACAAGCGGAAAATATCATTCACCTGTTTACACTGACAAATCTTGGCGCACCGGCAGCGTTTAAATACTTTGATACAACAATTGACCGCAAACGCTACACAAGCACAAAAGAAGTTCTGGACGCAACACTGATTCATCAATCAATTACAGGCCTTTATGAAACGAGCATTGATCTGTCACAACTGGGAGGCGATTGAATTGACACTAAAGGGATCCAGAAGCGGCAACACGCTAATCAATAAAAAAACGCTGTGCGGTTAAAGGGCACAGCGTTTTTTGTGTATGAATCGAAAAAGAGAACAGATCGCAGGTCTCAAAAATCGAGCGTAAAGGGCTGAT

>Sequence #3 – sgRNA::GFP expression cassette:

TGCTGTCCAGACTGTCCGCTGTGTAAAAAAAAGGAATAAAGGGGGGTTGACATTATTTTACTGATATGTATAATATAATTTGTATAAGAAAATG**TCTGTTAGTGGAGAGGGTGA**GTTTTAGAGCTAGAAATAGCAAGTTAAAATAAGGCTAGTCCGTTATCAACTTGAAAAAGTGGCACCGAGTCGGTGCTTTTCAATAAAAAAACGCTGTGCGGTTAAAGGGCACAGCGTTTTTTTGTGTATGTCGATTCACAAAAATAGGCACACGAAAAAC
